# *Psychrobacter halotolerans* sp. nov., a halotolerant plant growth-promoting bacterium that enhances lettuce tolerance to salt stress

**DOI:** 10.3389/fmicb.2026.1856557

**Published:** 2026-06-29

**Authors:** Patricia Sánchez, Fernando Martínez-Checa, Francisco Palma, Inmaculada Llamas, Inmaculada Sampedro

**Affiliations:** 1 Department of Microbiology, Faculty of Pharmacy, University of Granada, Granada, Spain; 2 Biomedical Research Centre (CIBM), Biotechnology Institute, Granada, Spain; 3 Department of Plant Physiology, Faculty of Science, University of Granada, Granada, Spain

**Keywords:** halotolerant bacteria, osmolytes, plant-growth promoting bacteria, *Psychrobacter*, salinity

## Abstract

A polyphasic taxonomic approach was used to characterize strain B38^T^, isolated from the phyllosphere of *Salicornia hispanica*. The strain is an aerobic, Gram-negative, halotolerant coccus–rod-shaped bacterium exhibiting optimal growth at 28 °C, pH 6–9, and up to 12% (w/v) NaCl. The genome of strain B38^T^ is 3.2 Mbp with a DNA G+C content of 43.81 mol%. Phylogenetic analysis based on the 16S rRNA gene sequences placed B38^T^ within the genus *Psychrobacter*. Although 16S rRNA gene sequencing showed high similarity to *P. alimentarius* (99.73%), genomic indices (ANI, AAI, and dDDH) remained below species delimitation thresholds. Chemotaxonomic features included C_18:1_ ω9c as the major fatty acid, Q-8 as the main quinone, and diphosphatidylglycerol as the major polar lipid. Consequently, strain B38^T^ (=CECT 31210; =LMG 33901) is proposed as *Psychrobacter halotolerans* sp. nov. Additionally, the plant growth-promoting (PGP) potential of the halotolerant strain B38^T^ was evaluated in lettuce under salt stress, alongside the salt-tolerance mechanisms induced by inoculation. Strain B38ᵀ significantly enhanced lettuce biomass, leaf area, and PSII quantum efficiency compared to non-inoculated controls. These benefits were linked to increased soluble sugars and phenolic compounds, modulation polyamine homeostasis, and reduced oxidative damage. Our results demonstrate that *P. halotolerans* B38ᵀ effectively mitigates salt stress in plants, representing a promising biotechnological tool for enhancing salinity tolerance in agricultural systems.

## 1 Introduction

Agriculture is the main source of global food production. The world population is estimated to reach around 10 billion by 2,100 (United Nations Department of Economic and Social Affairs, Population Division, 2022), making it necessary to increase food production to overcome the future increase food demand of 35–56% (van Dijk et al., 2021). Crops are exposed to a wide range of biotic stresses (such as microorganisms, nematodes and herbivores) and abiotic stresses (including salinity, high temperatures, drought and heavy metal toxicity) that adversely affect plant growth and yield (Majhi et al., 2025). Traditionally, fertilizers and pesticides have been the most widely used approaches to increase plant productivity. However, the overuse of these chemical methods has raised significant concerns, including the emergence of resistant pathogens, environmental contamination, soil deterioration and soil salinity (Tripathi et al., 2020).

Salinity is one of the major abiotic stresses limiting global agricultural productivity. Currently, approximately 20% of global arable land and 33% of irrigated land are affected by salt accumulation (Xie et al., 2024). Consequently, the development of saline soil-based agriculture has been prioritized. Nevertheless, most conventional crops are moderately sensitive to salinity (Machado and Serralheiro, 2017), including lettuce (*Lactuca sativa* L.). Lettuce is an economically significant crop with widespread global consumption and provides essential nutritional contribution to the human diet (Hassan et al., 2021; Zuzunaga-Rosas et al., 2024). Despite its importance, lettuce exhibits low salt tolerance, with an electrical conductivity threshold of only 2 dS/m (de Souza Lemos-Neto et al., 2026).

Salt stress negatively impacts both the growth and physiological parameters of lettuce, leading to significant reductions in plant height, number of leaves, and chlorophyll content. In addition, salinity disrupts nutrient homeostasis, particularly affecting the uptake of essential macronutrients such as nitrogen, phosphorus and potassium (Babaousmail et al., 2022; Sardar et al., 2023; Zuzunaga-Rosas et al., 2024). These physiological constraints represent a major challenge for commercial lettuce production in many agricultural regions.

At the mechanistic level, salinity imposes immediate osmotic stress, which restricts water uptake, and subsequent ion toxicity that interferes with nutrient absorption. In addition, salt stress induces oxidative stress through the accumulation of reactive oxygen species (ROS), which degrade various cellular components. These stress factors result in physiological and biochemical changes in plants, including inhibited seed germination, stomatal closure, reduced photosynthetic activity, and impaired leaf development, ultimately resulting in diminished plant growth and yield (Peng et al., 2023).

Plants have developed different tolerance mechanisms to cope with salt stress, including the production of enzymes and molecules that mitigate oxidative damage by detoxifying ROS, as well as the accumulation of low-molecular-weight organic compounds to reduce osmotic stress (Ilangumaran and Smith, 2017). Polyamines are nitrogenous, polycationic compounds present in plants, being putrescine, spermidine and spermine the most common type of polyamines in plants (Liu et al., 2025). Phenolic compounds are secondary metabolites produced by plants that exhibit protective effects against stress through ROS neutralization (Ray et al., 2024). Additionally, the synthesis and accumulation of compatible solutes, such as sugars in the cytosol, play an essential role in maintaining osmotic balance and water uptake under saline conditions (Kang et al., 2015; Ilangumaran and Smith, 2017).

Plant growth-promoting bacteria (PGPB) are non-pathogenic microorganisms that inhabit soil or plant tissue and have become an important alternative for sustainable agricultural development (Peng et al., 2023). PGPB enhance plant growth and productivity and improve tolerance to abiotic and biotic stresses through direct and indirect mechanisms, such as nitrogen fixation, phosphate solubilization, siderophore production and the secretion of lytic enzymes (Patel et al., 2024). Additionally, PGPB stimulate the synthesis and accumulation of protective compounds such as compatible solutes and antioxidant compounds (Kang et al., 2015; Ilangumaran and Smith, 2017; Xie et al., 2024; Sánchez et al., 2025). Recently, the isolation of halotolerant PGPB from saline environments has emerged as an effective strategy to help plants overcome salt stress because of the ability of these strains to survive across a wide range of salt concentrations.

Regarding the genus *Psychrobacter*, some species have been reported to promote plant growth (Ma et al., 2009; Ma et al., 2010). This growth-promoting ability has been attributed to the production of phytohormones, volatile organic compounds (Borker et al., 2024) and siderophores (Sinha et al., 2019). Additionally, some *Psychrobacter* species have been reported to attenuate pathogen virulence through quorum quenching (QQ) mechanisms (Packiavathy et al., 2021; Reina et al., 2021). The genus *Psychrobacter* comprises 47 species of aerobic, non-motile, Gram-negative coccobacilli capable of growing at temperatures ranging from −10 to 37 °C (Bakermans et al., 2006; Bowman, 2006). Members of this genus have been isolated from diverse natural environments, including Antarctic soils, sea ice, deep-sea ecosystems, and Pacific Ocean seawater (Bozal et al., 2003), and they exhibit high tolerance to salinity (Romanenko et al., 2002).

The aims of this study were (i) to perform a taxonomic characterization of strain B38^T^, isolated from the phyllosphere of *Salicornia hispanica* collected from El Saladar de El Margen, Cúllar, Granada (southern Spain) and (ii) to evaluate its potential to enhance the salt tolerance in lettuce plants and to analyse the plant responses induced by bacterial inoculation under salt stress.

## 2 Materials and methods

### 2.1 Bacterial strains, media and culture conditions

Strain B38^T^ was isolated from the phyllosphere of *Salicornia hispanica* collected in El Saladar de El Margen (Cúllar, Granada, Spain; 37° 38′50.6”N, 2° 37′22.2” W). Sampling was conducted at four distinct sites differing in salinity levels (0.5 to 1.6%), absence of surface water and sparse vegetation (site 4, exhibited the highest salinity). Samples included water, soil and halophytes.

For the isolation of plant-associated bacteria, whole plants as well as separated aerial and root tissues were processed. Plant material was gently washed in sterile saline solution, and aliquots of the wash and macerated tissues were plated onto tryptone soy agar (TSA) medium supplemented with 2 and 5% (w/v) NaCl. Plates were incubated at 28 °C for 7 days. A total of 42 morphologically distinct bacterial isolates were obtained and purified. These isolated were screened for halotolerance and for *in vitro* PGP traits (see above). Strain B38^T^ was selected for further study based on its performance in these assays. Notably, it was isolated from Site 4, the location with the highest salinity, supporting its adaptation to extreme saline conditions.

*Psychrobacter alimentarius* JG 100^T^ (DSM 16065), *P. vallis* CMS 39^T^ (DSM 15337) and *P. aquaticus* CMS 56^T^ (DSM 15339) were used for taxonomic identification. All of them were routinely grown in Luria Bertani (LB) media at 28 °C and rotary shaker at 120 rpm.

### 2.2 Phylogenetic 16S rRNA gene analysis

Genomic DNA of strain B38^T^ was extracted using the X-DNA Purification Kit (Xtrem Biotech S. L., Granada, Spain). The 16S rRNA gene was amplified using the universal bacterial primers 16F27 and 16R1488. The PCR product was purified and cloned into the pGEM®-T vector (Promega). Direct sequencing of the amplified DNA was performed, and the resulting sequence was compared with reference 16S rRNA gene sequences from GenBank and EMBL databases via the NCBI Genome Database using EzBioCloud server (Yoon et al., 2017).

Phylogenetic analysis was conducted using the Molecular Evolutionary Genetics Analysis (MEGA) software version X (Kumar et al., 2018), incorporating multiple sequence alignments with CLUSTAL OMEGA (Sievers et al., 2011). Evolutionary distances and clustering were determined using the neighbor-joining and maximum-likelihood methods. Cluster stability was assessed through bootstrap analysis with 1,000 replications.

### 2.3 Multilocus sequence analysis

To construct a more robust phylogenetic tree, a multigene approach was used. MLSA was conducted by concatenating the sequences of four housekeeping genes (16S rRNA, *gyrB*, *rpoD*, and *rpoB*). Housekeeping gene sequences from other *Psychrobacter* species were retrieved from the genomes deposited in NCBI GenBank database. Multiple sequence alignment of the concatenated gene sequences was performed using the MUSCLE algorithm embedded in MEGA X, followed by manual verification to identify and correct alignment errors.

A phylogenetic tree was generated using the neighbor-joining (NJ) algorithm, with 1,000 bootstrap replicates to assess the robustness of the branching.

### 2.4 Nucleic acid extraction and whole-genome sequencing, assembly and annotation

Genomic DNA of strain B38^T^, which was extracted as described above, was sequenced by the Illumina MiSeq methodology at the STAB VIDA facility (Caparica, Portugal) with 2 × 150-bp paired-end reads. The reads were trimmed using software tools implemented in the BBMap project^1^ (Bushnell, 2016) to remove the adapters and low-quality bases and *de novo* assembled using SPADES v3.11.1 (Bankevich et al., 2012). CheckM v1.0.18 (Parks et al., 2015) and Quast v5.0.2 (Gurevich et al., 2013) were used for assembly quality checked. The genome of strain B38^T^ was annotated using RASTtk v1.073 (Aziz et al., 2008; Overbeek et al., 2014; Brettin et al., 2015) and deposited in GenBank/EMBL/ DDBJ under the accession number JBDKWD000000000.

### 2.5 *In silico* ANI, AAI and DDH

Average nucleotide identity (ANI) was determined by using OrthoANI software (Lee et al., 2016). Average amino acid identity (AAI) was calculated from protein sequences using the AAI online tool available on the Kostas Lab website^2^, applying the two-way AAI algorithm to ensure accurate bidirectional assessment. For digital DNA–DNA hybridization (dDDH), values were calculated using the BLAST+ algorithm via the DSMZ Genome-to-Genome Distance Calculator (GGDC 3.0) web service^2^ (Meier-Kolthoff et al., 2013; Meier-Kolthoff et al., 2022). The results presented in this study are based on the recommended Formula 2 (identities/HSP length), which is independent of genome length, ensuring robustness even when using incomplete draft genomes. The results of the strain described in this study were additionally validated using the Type (Strain) Genome Server [TYGS (Meier-Kolthoff et al., 2022)] in January 2025.

### 2.6 Analysis of the core orthologous genes

A core genome analysis of strain B38^T^ and 16 closest related bacteria based on their 16S rRNA percentage of similarity for which their genome was available, downloaded from the NCBI RefSeq database, was also performed using Bacterial Pan Genome Analysis (BPGA) software (Chaudhari et al., 2016) with the default parameters. After obtaining the core of these 16 bacterial genomes, all protein orthologs belonging to the core genome were concatenated and aligned by MAFFT (Katoh and Standley, 2013). A phylogenomic tree of the core genes of the species was then constructed using MEGA X software according to the maximum likelihood method, incorporating 1,000 bootstrap replicates to evaluate the robustness of the branching.

### 2.7 Phenotypic and chemotaxonomic characterization

Colony morphology of strain B38^T^ was observed on LB agar after 48 h of incubation at 28 °C. Oxidase and catalase activities were determined. Salinity tolerance and optimal growth conditions of strain B38^T^ were assessed at 28 °C on LB agar plates supplemented with 0; 0.5; 2.5; 5; 7.5; 12 and 25% (w/v) of NaCl at pH 7. The pH growth range and optimum pH were analysed on LB agar plates, testing from 4 to 10 pH unit intervals. The temperature range and optimum temperature were determined on LB agar plates incubated at 4; 10; 15; 20; 25; 30 and 37 °C. In all cases, media contained 1% (w/v) of NaCl.

Phenotypic characteristics of strain B38^T^ related with *in vitro* PGP activities and rhizosphere competence were evaluated including amylase (Barrow and Feltham, 1993), caseinase (Barrow and Feltham, 1993), cellulase (Tasse et al., 2010), acid phosphatase (Baird-Parker, 1963), alkaline phosphatase (Pikovskaya, 1948), phytase (Hosseinkhani and Hosseinkhani, 2009), gelatinase (Tindall et al., 2007), lecithinase (Larpent and Larpent-Gourgand, 1957) and chitinase activities; hydrolysis of Tween 20 and Tween 80 (Sierra, 1957), and production of siderophores (Alexander and Zuberer, 1991).

Additional biochemical tests were performed using cells of B38^T^ grown under optimal conditions with API 20NE, API 50CH, API ZYM and BIOLOG GEN III systems, according to the manufacturer’s instructions. In addition, DNase activity (Jeffries et al., 1957) was detected.

Cellular fatty acids composition was analysed at the Spanish Type Culture Collection (CECT) in Valencia, Spain, following the procedures described in the Microbial Identification System Operating Manual (MIDI, 2008). For this, cell biomass of strain B38^T^ was obtained after growing for 24 h in LB broth at 28 °C with 1% (w/v) NaCl. The analysis of polar lipids and respiratory quinones of strain B38^T^ was performed by the Identification Service at DSMZ in Braunschweig, Germany.

### 2.8 Plant material and growing conditions

Seeds of *Lactuca sativa* L. var. Maravilla de Verano were germinated and cultivated for 15 days in trays divided into cells (3 cm × 3 cm × 10 cm) at Abonos Bolivar S. L. (Granada, Spain). Subsequently, seedlings were transferred into individual pots of about 300 mL containing a sterilized vermiculite-perlite mixture (3:1) and maintained in a controlled-environment growth chamber: a 16/8 h light–dark cycle, 25/18 °C day-night temperature, relative humidity 60–70% and photosynthetic photon flux density of 450 μmol m^−2^ s^−1^. The experimental treatments were the following: (i) non-inoculated control plants, (ii) plants inoculated with strain B38^T^, (iii) plants treated with 100 mM NaCl and (iv) plants treated with 100 mM NaCl and inoculated with strain B38^T^. For PGPB treatment, 250 μL of bacterial suspension (10^8^ CFU mL^−1^) was applied three times over a seven-day period. Control plants were treated with the same volume of sterile water. Salt stress was applied after the bacterial inoculation period by irrigating with Hoagland Solution (Atero-Calvo et al., 2024a) containing 0 and 100 mM NaCl at two-day intervals for 10 days. The electrical conductivity of 0 mM and 100 mM NaCl Hoagland solutions was monitored throughout the 10-day experiment, with values of 2.64 dS m^−1^ and 14.2 dS m^−1^, respectively. Each treatment was composed of twelve plants.

Chlorophyll fluorescence was measured using a portable chlorophyll fluorescence meter (Handy PEA, Hansatech, UK) after 30 min of dark adaptation (Atero-Calvo et al., 2024b), using a special leaf clip holder placed on fully expanded leaves at the mid-stem position of six plants per treatment. The parameter employed to measure photosynthetic activity was maximum quantum efficiency of photosystem II (PSII) (Fv/Fm, where Fv is variable fluorescence calculated as Fv = Fm − Fo, being Fm the maximum fluorescence and Fo the initial fluorescence). The same plants were used for measurements of plant length, plant dry weight, and leaf area. Leaf area was calculated using ImageJ software (Schneider et al., 2012). Three biological replicates were prepared for each treatment from the remaining plants, with each replicate consisting of leaves from two randomly selected plants. Each biological replicate was frozen in liquid nitrogen, pulverized, and stored at −80 °C. Subsequently, the material was lyophilized and used for biochemical determinations.

### 2.9 Total free phenols content

Phenolic compounds were determined following the protocol described by Singleton et al. (1999) with minor modifications. The lyophilized plant material (1:100, w/v) was extracted using acetone:water extraction medium (80:20, v/v) in darkness for 30 min at 4 °C and centrifuged at 8,000 ×*g* for 10 min at 4 °C. The total free phenols content of the supernatant was determined by the Folin–Ciocalteu method. Results were expressed as mg of gallic acid per g of dry weight.

### 2.10 Measurement of lipid peroxidation

Lipid peroxidation was determined as malondialdehyde (MDA) following the protocol described by Heath and Packer (1968), with some modifications. Lyophilized plant material was homogenized (1:85, w/v) in 20% (w/v) trichloroacetic acid (TCA) and 4% (w/v) butylated hydroxytoluene. The homogenate was centrifuged at 10,000 ×*g* for 15 min at 4 °C. MDA content of the supernatant was determined by a colorimetric reaction using 0.5% (w/v) thiobarbituric acid (TBA) in 20% (w/v) TCA. The mixture was heated at 95 °C for 30 min, cooled immediately in ice to stop the reaction and centrifuged at 5,000 ×*g* for 10 min at 4 °C. Absorbance of the supernatant was estimated at 532 nm and at 600 nm for non-specific absorbance. Data were calculated according to a calibration curve obtain with MDA. Results were expressed as mg of MDA per g of dry weight.

### 2.11 Total soluble sugars content

Total soluble sugars were determined following the protocol described by Palma et al. (2014). Total soluble sugars were extracted from lyophilized plant material (1:100, w/v) using ethanol:chloroform:water extraction medium (12:5:1, v/v/v) and centrifuged at 5,000 ×*g* for 10 min at 4 °C. The supernatant was separated into aqueous and chloroform phases by the addition of chloroform and water. Soluble carbohydrates were determined from the aqueous phase by mixing the sample supernatant with anthrone reagent at a 1:19 (v/v) ratio. The mixture was heated at 95 °C for 10 min, cooled immediately in ice to stop the reaction and centrifuged at 5,000 ×*g* for 5 min at 4 °C. Absorbance of the samples was estimated at 625 nm. A standard curve prepared with glucose was used to estimate the concentration of total soluble sugars. Results were expressed as mg of glucose per g of dry weight.

### 2.12 Putrescine, spermidine and spermine content

Polyamines were determined following the protocol described by Palma et al. (2015). Polyamines were extracted from lyophilized plant material (1:70, w/v) using 5% (v/v) cold perchloric acid and 1,7-diaminoheptane (60 nmol mL^−1^) as internal standard and incubated for 24 h at 4 °C. The mixture was centrifuged at 5,000 ×*g* for 5 min at 4 °C. The supernatant was used to determine free polyamines. Polyamines were derivatized under alkaline conditions with 1% (w/v) dansyl chloride in acetone and incubated in darkness at room temperature overnight. Dansylpolyamines were extracted with toluene, evaporated to dryness under an air stream, and redissolved in acetonitrile. Polyamine analysis was performed by HPLC using an Agilent 1,260 Infinity system equipped with a C18 column (Varian, 5 μm, 250 mm × 4.6 mm). The flow rate was 1.5 mL min^−1^, and separation was achieved using a water (A) and acetonitrile (B) gradient. The gradient profile was applied as follow (t (min); %A): (0; 30%), (4.5; 30%), (9; 0%), (14; 0%), (15; 30%). Finally, fluorescence detection was carried out at excitation and emission wavelengths of 415 and 510 nm, respectively. Results were expressed as μg per g of dry weight.

### 2.13 Statistical analysis

Statistical analyses were conducted using Statistical Package for the Social Sciences (SPSS), version 28.0.0.^3^ The experiment followed a completely randomized factorial design with two factors: bacterial inoculation (non-inoculated and inoculated with strain B38^T^) and salt stress (0 and 100 mM NaCl), resulting in four treatments. Each treatment included 12 independent plants (*n* = 12). For physiological and morphological measurements, six plants per treatment were randomly selected and analyzed individually. The remaining plants were used for biochemical analyses, for which three biological replicates per treatment were established, each consisting of pooled material from two plants (Supplementary Figure S1). Data normality was evaluated using the Shapiro–Wilk test. Finally, ANOVA and post-hoc Tukey analyses were used to assess the effects of each treatment.

### 2.14 Data availability

Strain B38^T^ can be obtained from the Spanish Collection of Type Cultures (CECT31210) and the BCCM/LMG Bacteria Collection (LMG33901). The sequence of the 16S rRNA gene of strain B38^T^ can be found under GenBank accession number GCA_039680845. The accession number for the whole-genome sequence of strain B38^T^ is JBDKWD000000000. The associated BioSample and BioProject accession numbers are SAMN41148270 and PRJNA1106776, respectively.

## 3 Results

### 3.1 Phylogenetic and MLSA analyses of strain B38^T^

**Figure 1:**
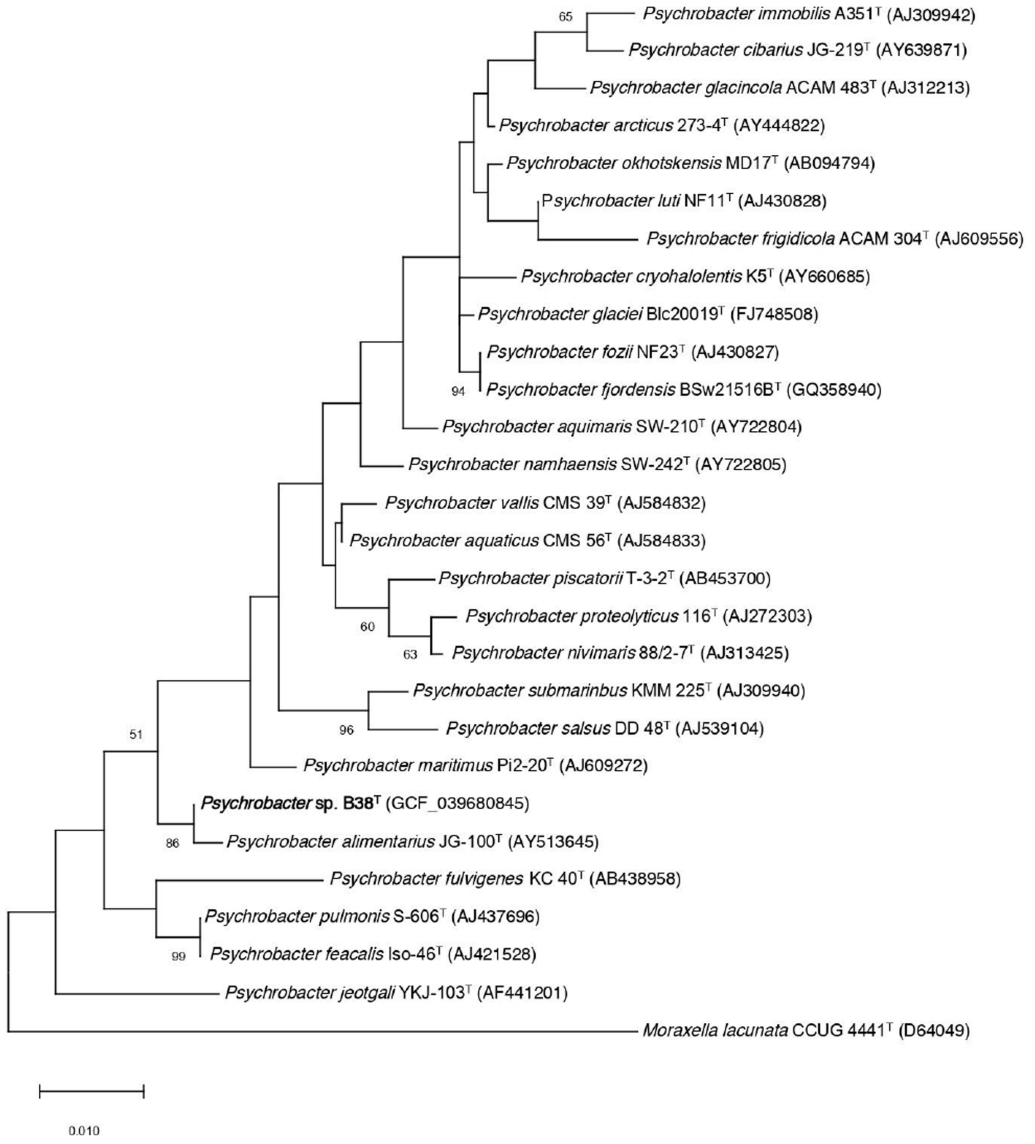
Phylogenetic position of the strain B38^T^ 16S rRNA gene sequence (bold) and its relationship with other related species by using the maximum likelihood algorithm. The GenBank/EMBL/DDBJ accession number of each sequence is shown in parenthesis. Bootstrap values are expressed as percentages of 1,000 replications, and those >50% are shown at branch points. Bar shows sequence divergence. Bar 0.010 substitutions per nucleotide position. *M. lacunata* CCUG 4441^T^ sequence was used as an outgroup.

The nearly complete 16S rRNA gene sequence (~1,500 bp) of strain B38^T^ was amplified by PCR and showed complete identity with the corresponding sequence obtained from its sequencing genome. Comparative sequence analysis revealed that strain B38^T^ shared the highest sequence identity with *Psychrobacter alimentarius* JG 100^T^ (99.73%), followed by *P. vallis* CMS 39^T^ (98.90%) and *P. aquaticus* CMS 56^T^ (98.56%). Sequence identities below 97% were observed with other *Psychrobacter* species. Phylogenetic analysis of its 16S rRNA gene sequence and other related strains, using the maximum-likelihood algorithm, confirmed that strain B38^T^ belongs to the genus *Psychrobacter* and clusters with *P. alimentarius* JG 100^T^, the species showing the highest sequence identity (Figure 1).

Furthermore, the MLSA resulting neighbor-joining tree derived from concatenated sequences of four housekeeping genes (16S rRNA, *gyrB*, *rpoD*, and *rpoB*) supported that B38^T^ and *P. alimentarius* JG 100^T^ form a robust cluster with a bootstrap value of 99% (Supplementary Figure S2).

### 3.2 Whole-genome sequencing and assembly

The draft genome of strain B38^T^ was manually curated, resulting in a total genome size of 3.2 Mbp. Assembly quality was assessed using the Quality Assessment Tool for Genome Assemblies (QUAST), which produced an N50 value of 421.7 Kb and an average sequencing coverage of 200X, demonstrating high assembly reliability. Genome annotation, performed with the Prokaryotic Genome Annotation Pipeline, PGAP (Tatusova et al., 2016), identified 2,621 protein-coding genes (PCGs). The assembled genome sequence has been deposited in the GenBank/EMBL/DDBJ database under the accession number GCA_039680845 and served as the basis for further analyses.

### 3.3 *In silico* G+C content, ANI, AAI and DDH calculations

The *in silico* analysis of the G+C content of the draft genome of strain B38^T^ revealed a value of 43.81%. Average nucleotide identity (ANI) values, calculated using OrthoANI, indicated that strain B38^T^ and phylogenetically related species shared values below the species delimitation threshold of 95–96% (Chun et al., 2018). Similarly, average amino acid identity (AAI) analysis demonstrates that the values between strain B38^T^ and related species were also below the species delimitation threshold of 95–96% (Konstantinidis and Tiedje, 2005) (Supplementary Table S1). Digital DNA–DNA hybridization (dDDH) analysis based on whole-genome sequences of strain B38^T^ and its closest related species also showed values below the 70% species delineation threshold (Supplementary Table S1).

### 3.4 Phylogenetic analysis of core orthologous proteins

**Figure 2:**
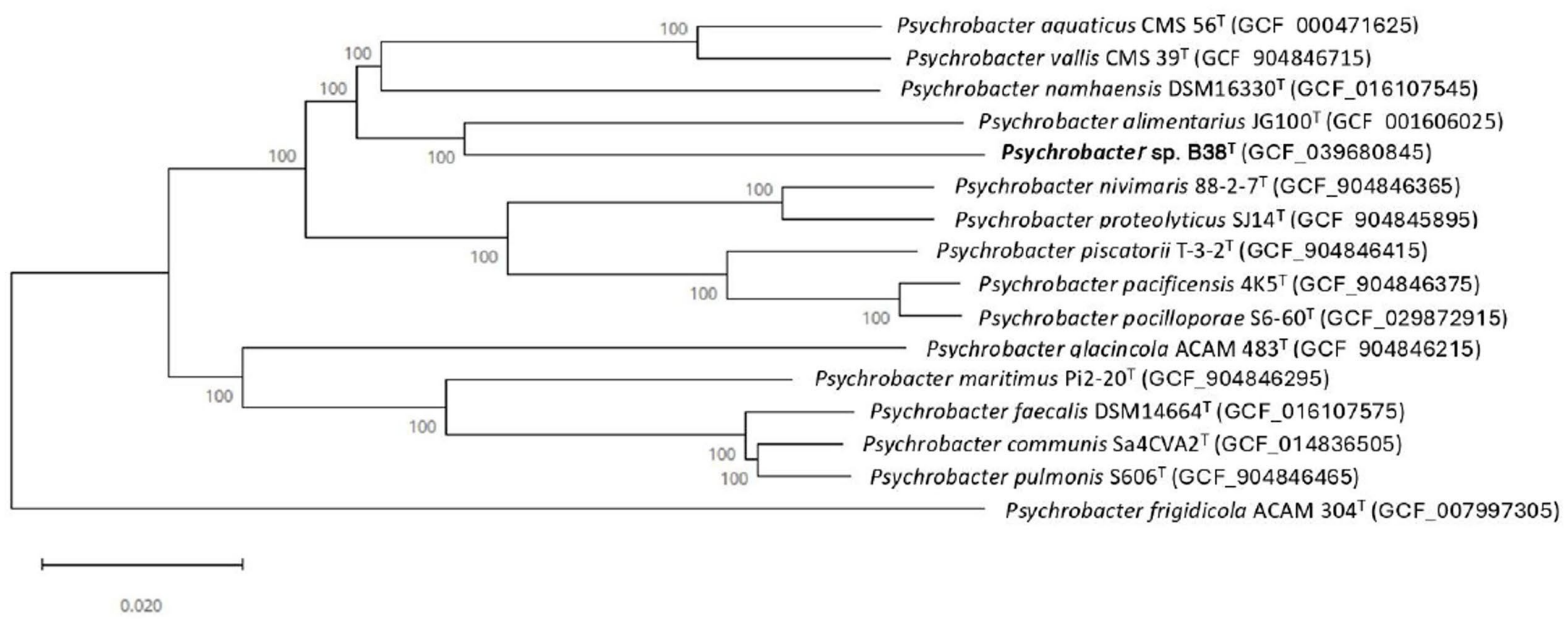
Tree constructed according to the maximum-likelihood method based on 1,510 core orthologous proteins of the strain B38^T^ (bold) and the available genomes of *Psychrobacter* related species. Bootstrap values are expressed as percentages of 1,000 replications, and those over 50% are shown at branch points. Bar 0.020 substitutions per nucleotide position.

A concatenated alignment of 1,510 core orthologous proteins from strain B38^T^ and related species within the genus *Psychrobacter* was employed to construct a maximum-likelihood phylogenetic tree. The resulting tree is presented in Figure 2.

### 3.5 Chemotaxonomic characteristics

The predominant cellular fatty acid of strain B38^T^ was C_18:1_ ω9c (64.58%) and summed feature 3 (contains C_16:1_ ω7c and/or C_16:1_ ω6c) (11.27%), while other fatty acids were detected in minor concentrations such as C_18:0_ (5.78%), C_16:0_ (4.31%), C_10:0_ (3.28%), C_17:1_ ω8c (2.34%) and C_12:0_-3OH (2.62%). The major polar lipid of strain B38^T^ was diphosphatidylglycerol. Other polar lipids present were phosphatidylethanolamine, phosphatidylglycerol, an unidentified glycolipid and an unidentified phospholipid (Supplementary Figure S3). The predominant respiratory quinone of strain B38^T^ was ubiquinone-8 (Q-8).

### 3.6 Morphological, physiological and biochemical characteristics

Cells of strain B38^T^ were Gram-negative, coccus-rod-shaped and motile (Supplementary Figure S4). After 48 h of incubation on LB agar plates, the strain forms small, creamy colonies. It was both catalase and oxidase positive. It was aerobe and exhibited growth within a temperature range from 4° C to 32° C, with optimal growth observed at 28° C. It grew over a pH range from 6 to 10, with an optimum between pH 6 and 9. Strain B38^T^ resulted to be halotolerant, growing in the presence of 0–12% (w/v) of NaCl, with optimal growth at 2.5% (w/v). Differential phenotypic characteristics distinguish this strain from its closely related species, *P. alimentarius* DMS 16065, *P. aquaticus* DSM 15339 and *P. vallis* DSM 15337, are listed in Table 1. Notably, strain B38^T^ did not assimilate D-arabitol, D-fructose, D-galacturonic acid, D-gluconic acid, D-glucuronic acid, D-mannitol, D-mannose, D-saccharic acid, D-salicin, glycerol, L-galactonic acid lactone, L-pyroglutamic acid, mucic acid, myo-inositol, N-acetyl-D-galactosamine, N-acetyl-D-glucosaminase, N-acetyl-D-mannosamine, N-acetyl-neuraminic acid, quinic acid, stachyose and β-methyl-D-glucoside unlike the related species. Furthermore, strain B38^T^ was β-glucosidase negative while the related species were positive. In addition, strain Β38^T^ was sensitive to lincomycin, while the other species were resistant. Other phenotypic traits of strain B38^T^ were provided in the species description and in Supplementary Table S2.

**Table 1:** Differential characteristic between strain B38^T^ with respect to its closest species of the *Psychrobacter* genus.

Characteristic	1	2	3	4
API 20NE tests				
Hydrolysis (β-glucosidase)	−	+	+	+
Reduction of nitrates to nitrites	−	−	+	+
Oxidation of (API 50 CH)				
D-arabinose	−	w	−	−
D-fucose	−	+	w	w
D-galactose	−	+	w	−
D-glucose	−	+	w	w
D-lactose (bovine origin)	−	w	−	−
D-mannose	−	+	w	w
D-melibiose	−	+	w	w
D-ribose	−	+	w	w
D-sorbitol	−	w	−	−
D-xylose	−	+	w	w
Gentiobiose	−	w	−	−
L-arabinose	−	+	w	w
L-rhamnose	−	w	−	−
L-xylose	−	w	−	−
N-acetylglucosamine	−	−	+	−
Enzymes (API ZYM)				
Cystine arylaminidase	−	+	−	−
Esterase (C4)	−	−	+	+
Napthol-AS-BI-phosphohydrolase	w	+	−	w
α-galactosidase	+	+	+	−
Assimilation of (biolog)				
Bromo-Succinic Acid	+	+	w	+
Citric acid	−	+	w	+
D-arabitol	−	+	+	+
D-aspartic acid	w	+	+	+
D-cellobiose	−	w	w	w
D-fructose	−	+	+	+
D-fructose 6-PO_4_	w	+	+	w
D-fucose	+	−	w	−
D-galactose	−	+	w	−
D-galacturonic acid	−	+	+	+
D-gluconic acid	−	+	+	+
D-glucose-6-PO_4_	w	+	+	+
D-glucuronic acid	−	+	+	+
D-lactic acid methyl esther	+	+	w	+
D-maltose	−	w	w	w
D-mannitol	−	+	+	+
D-mannose	−	+	+	+
D-melibiose	−	+	+	w
D-raffinose	−	+	w	w
D-saccharic acid	−	+	+	+
D-salicin	−	+	+	+
D-serine	w	+	+	+
D-sorbitol	−	−	+	W
D-trehalose	−	w	w	w
D-turanose	−	+	+	w
Formic Acid	w	+	+	+
Gelatin	w	+	+	w
Gentiobiose	−	w	+	w
Glucuronamide	−	+	+	+
Glycerol	−	+	+	+
Inosine	−	+	w	−
L-fucose	+	+	−	−
L-galactonic acid lactone	−	+	+	+
L-pyroglutamic acid	−	+	+	+
Methyl piruvate	+	+	w	+
Mucic acid	−	+	+	+
*myo*-Inositol	−	+	+	+
N-acetyl-D-galactosamine	−	+	+	+
N-acetyl-D-glucosaminase	−	+	+	+
N-acetyl-D-mannosamine	−	+	+	+
N-acetyl-neuraminic acid	−	+	+	+
Pectin	w	+	+	−
Quinic acid	−	+	+	+
Stachyose	−	+	+	w
Sucrose	−	+	w	w
α-D-glucose	−	+	w	−
α-D-lactose	−	+	w	w
β-Hydroxy-Phenylacetic Acid	−	+	w	−
β-methyl-D-glucoside	−	+	+	+
Sensitivity to (biolog)				
1% (w/v) sodium lactate	+	+	+	−
8% (w/v) NaCl	+	+	−	+
D-serine	−	+	w	−
Guanidine HCL	−	+	−	−
Lincomycin	+	−	−	−
Nalidixic Acid	+	−	w	w
pH 5	+	−	−	−
Tetrazolium violet	+	+	−	−
DNA G+C content (*in silico*) (mol%)	43.81	44.00	43.60	44.50
Main Quinone	Q8	Q8	Q8	Q8

Strains: 1, B38^T^; 2, *P. alimentarius* DSM 16065; 3, *P. aquaticus* DSM 15339; 4, *P. vallis* DSM 15337. All data were obtained from this study. Only the most relevant results are shown. The complete results can be found in Supplementary Table S2. For assimilation and oxidation of carbon compounds: +, positive; −, negative; w, weakly positive. For sensitivity: +, insensitive (can grow in its presence); −, sensitive (cannot grow in is presence).

Regarding PGP traits, strain B38^T^ resulted positive activity for acid phosphatase, DNase, phytase and hydrolysis of Tween 20 and Tween 80, and it also produced siderophores and had *α* hemolysin activity. However, it did not present amylase, caseinase, cellulase, chitinase, gelatinase, alkaline phosphatase and lecithinase activities (Supplementary Table S3).

### 3.7 Effect of bacterial inoculation on lettuce growth under non-saline and saline conditions

**Figure 3:**
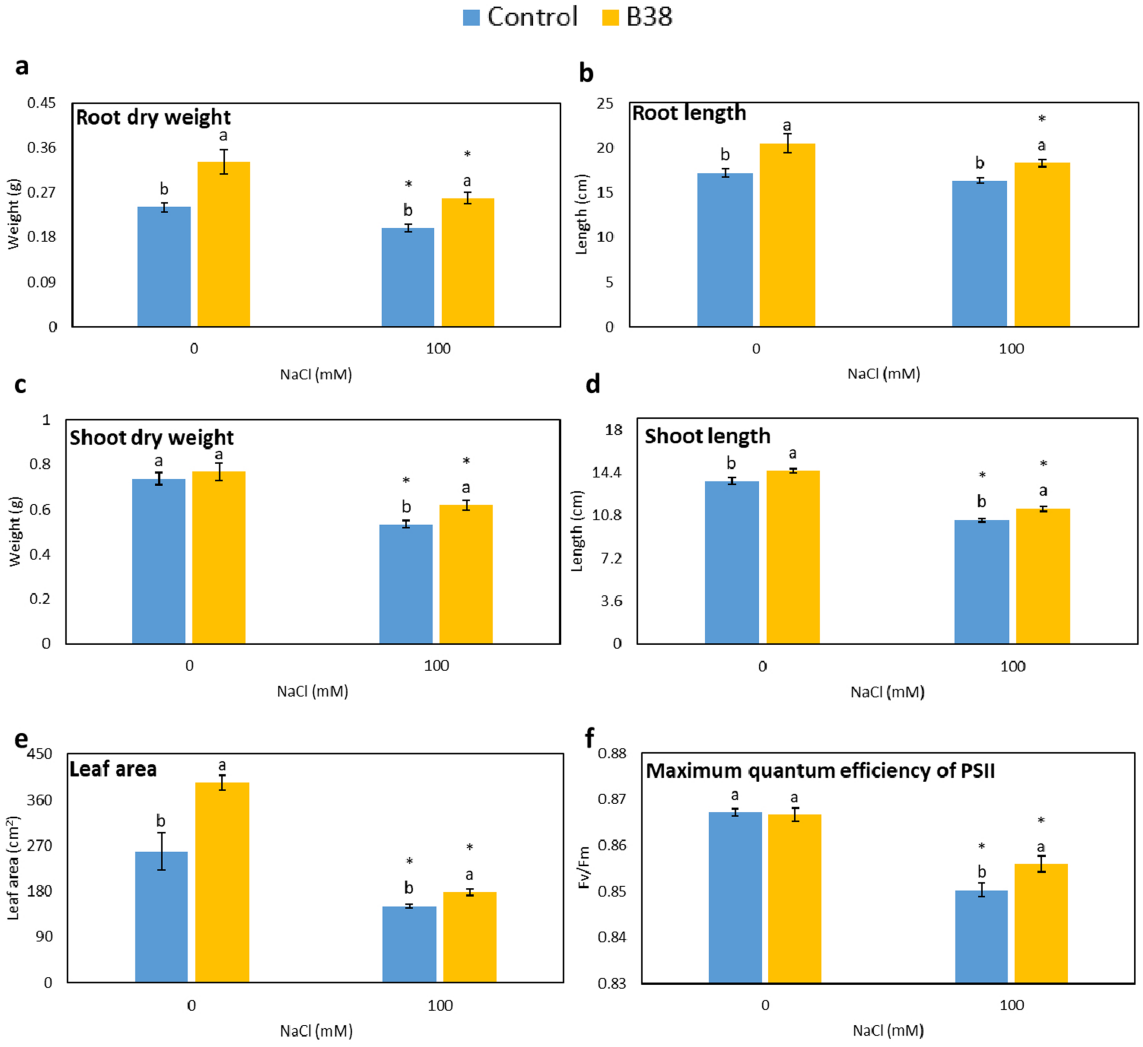
Effects of PGPB inoculation on root **(a)** and shoot **(c)** dry weight, root **(b)** and shoot **(d)** length, leaf area **(e)**, and maximum quantum efficiency of PSII **(f)** on lettuce plants under 0 and 100 mM NaCl. Data presented are means ± SE (*n* = 6). Different letters indicate significant differences between treatments (control vs. B38^T^) at the same salinity level (*p* < 0.05), whereas asterisks indicate significant differences between salinity levels (0 and 100 mM NaCl) within the same treatment (*p* < 0.05), according to Tukey’s test.

The adverse effect of salinity on lettuce plants is shown in Figure 3, where both non-inoculated and B38-inoculated plants showed significant reductions in growth parameters and photosynthetic efficiency at 100 mM NaCl compared to 0 mM NaCl. Nevertheless, inoculation with strain B38^T^ showed a positive effect on lettuce growth 10 days after inoculationin the absence of salt stress, resulting in increases in root dry weight (33.3%; Figure 3a), root length (18.1%; Figure 3b), shoot length (6.4%; Figure 3d) and leaf area (44.9%; Figure 3e) compared to non-inoculated plants. In contrast, no significant differences were observed in shoot dry weight or in the maximum quantum efficiency of PSII under this condition.

The PGP activity of B38^T^ in lettuce plants was also evaluated under saline stress, where the inoculation with this strain significantly increased all the measured parameters compared to non-inoculated plants, including root dry weight (30.3%), root length (12.2%), shoot dry weight (16.3%), shoot length (9.3%), leaf area (18.3%) and maximum quantum efficiency of PSII (0.67%) (Figure 3).

### 3.8 Effects of bacterial inoculation on osmolyte accumulation in lettuce leaves under saline and non-saline conditions

**Figure 4:**
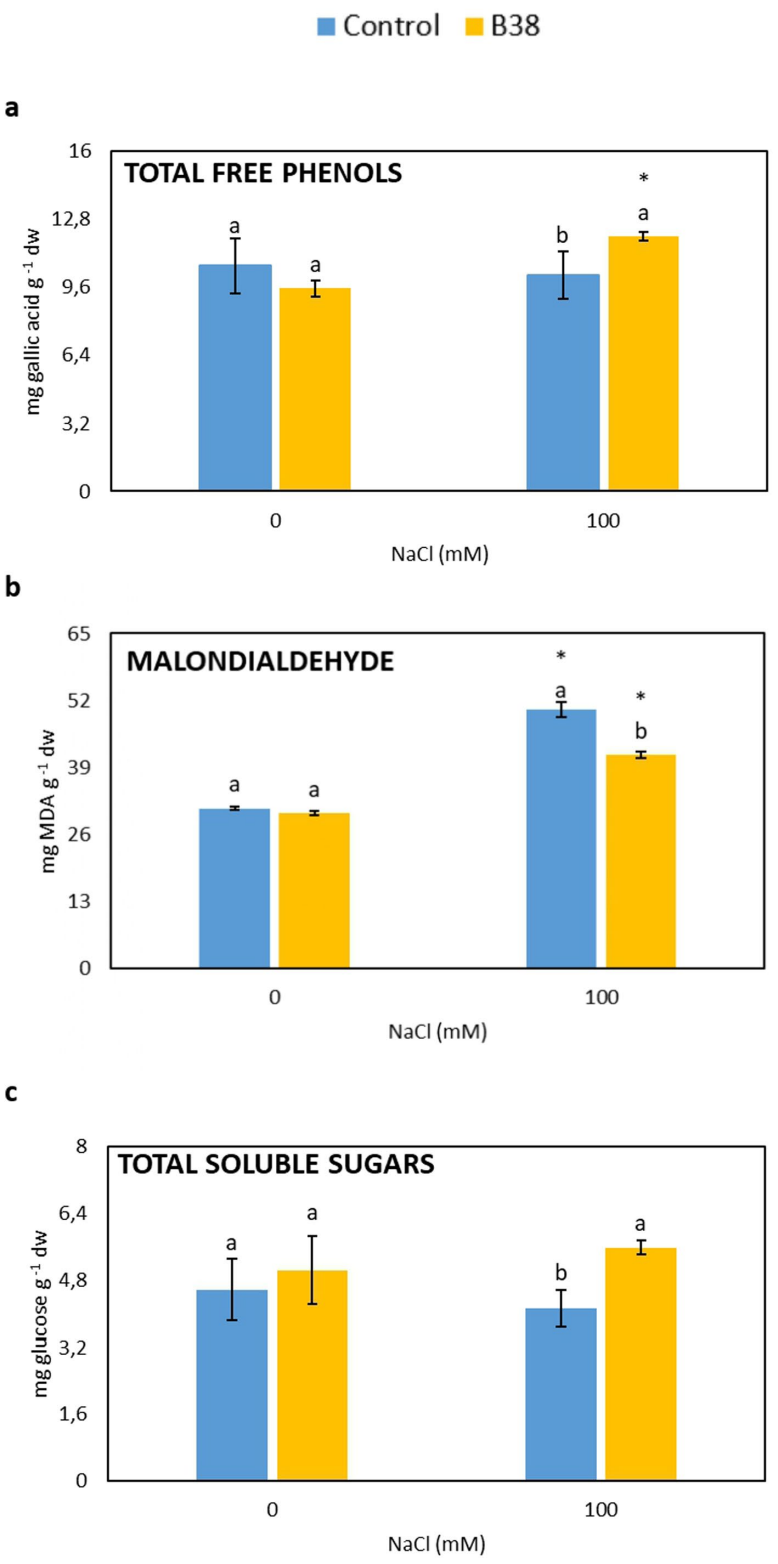
Changes in the content of total free phenols **(a)**, malondialdehyde **(b)**, and total soluble sugars **(c)** in lettuce plants treated with 0 and 100 mM NaCl and inoculated with B38^T^ strain. Data presented are means ± SE (*n* = 3). Different letters indicate significant differences between treatments (control vs. B38^T^) at the same salinity level (*p* < 0.05), whereas asterisks indicate significant differences between salinity levels (0 and 100 mM NaCl) within the same treatment (*p* < 0.05), according to Tukey’s test.

Considering the previous results observed in this study, we evaluated the role of strain B38^T^ on stress-protective metabolites accumulation in lettuce plant under non-salt stress and salt stress conditions. Under non-saline conditions, total free phenolic content did not differ significantly between control plants and those inoculated with the PGP strain. In contrast, under saline conditions, plants inoculated with strain B38^T^ showed an approximately 20% increase in total phenolic content compared with non-inoculated plants. In addition, B38^T^-inoculated plants exhibited higher phenolic compounds levels at 100 mM NaCl than under non-saline conditions, whereas no significant differences were detected between control plants grown at 0 and 100 mM NaCl (Figure 4a). Regarding MDA content, no significant differences among treatments were observed under non-saline conditions. However, at 100 mM NaCl, inoculation with strain B38^T^ reduced MDA levels around 20% compared with non-inoculated plants. In both control and inoculated plants, MDA content increased under saline conditions relative to non-saline conditions, indicating enhanced lipid peroxidation by salinity (Figure 4b). Concerning total soluble sugars, no significant differences among treatments were observed under non-saline conditions. However, under saline stress, soluble sugars content increased by 35.4% in B38^T^-inoculated plants respect to non-inoculated plant (Figure 4c).

**Figure 5:**
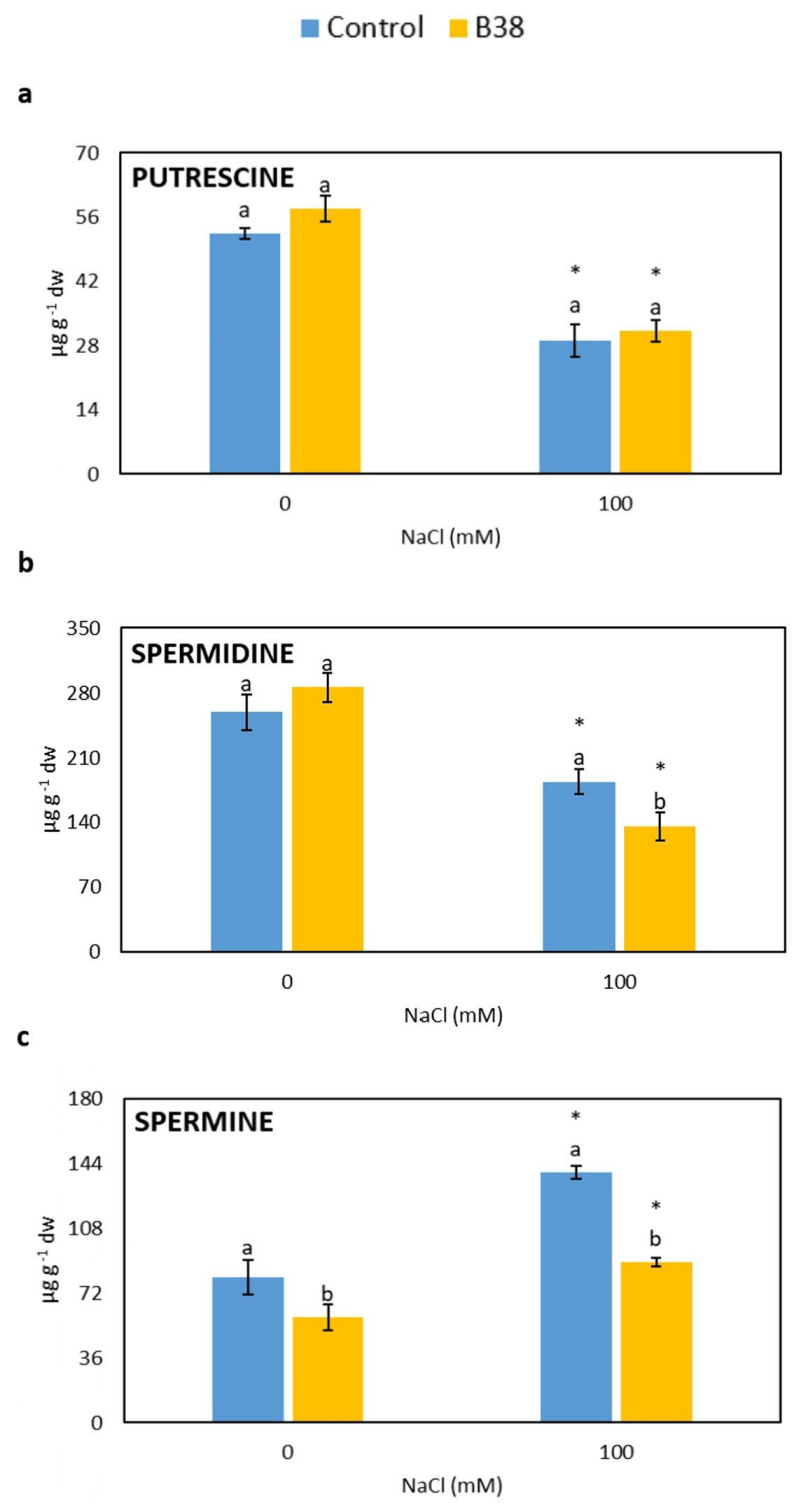
Changes in the content of putrescine **(a)**, spermidine **(b)**, and spermine **(c)** in lettuce plants treated with 0 and 100 mM NaCl and inoculated with B38^T^ strain. Data presented are means ± SE (*n* = 3). Different letters indicate significant differences between treatments (control vs. B38^T^) at the same salinity level (*p* < 0.05), whereas asterisks indicate significant differences between salinity levels (0 and 100 mM NaCl) within the same treatment (*p* < 0.05), according to Tukey’s test.

In the present study, the accumulation of the main polyamines described in plants, putrescine, spermidine, and spermine, were also analysed (Figure 5). Putrescine content showed no significant changes between control and B38^T^-inoculated plants under either non-saline or saline conditions. However, both treatments showed a reduction in putrescine content under saline conditions compared with non-saline conditions (Figure 5a). Spermidine concentration under non-saline conditions did not differ between B38^T^-inoculated plants and non-inoculated plants. Nevertheless, at 100 mM NaCl, spermidine levels decreased by 26.1% in B38^T^-inoculated plants compared with control plants. In both treatments, spermidine concentration decreased under saline stress compared with non-saline conditions (Figure 5b). Spermine concentration was lower in B38^T^-inoculated plants than in non-inoculated plants under both non-saline and saline conditions. Under no-saline conditions, B38^T^ inoculation reduced spermine content by 23.5%, whereas under saline stress the reduction reached 35.8%. Despite this, spermine levels increased under saline conditions relative to non-saline conditions in both treatments (Figure 5c).

## 4 Discussion

Salinity stress is one of the most significant abiotic factors reducing crop yields worldwide. The agricultural sector represents the largest source of income for a major part of the global population, and soil salinity constitutes a serious problem, particularly in irrigated soils. Therefore, research aimed at alleviating salinity stress is critically important to boost crop productivity and meet the increasing global food demand. In this context, PGPB can be considered as an eco-friendly and cost-effective strategy to combat salinity stress, providing an alternative to the intensive use of chemicals and fertilizers (Bhise and Dandge, 2019).

In this study, strain B38^T^, isolated from the phyllosphere of *Salicornia hispanica*, was first taxonomically characterized. Subsequently, its protective effect against salt stress in lettuce plants and the plant responses induced by bacterial inoculation were evaluated.

Genomic analyses revealed an *in silico* G+C content of 43.81 mol%, which falls within the range of 41–51 mol% described for the genus *Psychrobacter* (Bozal et al., 2003; Bowman, 2006). OrthoANI and AAI values calculated between strain B38^T^ and its closest related species, including *P. alimentarius* JG 100^T^, *P. vallis* CMS 39^T^ and *P. aquaticus* CMS 56^T^, were below the established delimitation threshold of 95–96% (Konstantinidis and Tiedje, 2005; Chun et al., 2018). Likewise, digital DNA–DNA hybridization values between strain B38^T^ and these related species were below the species delimitation threshold of 70% (Goris et al., 2007). Furthermore, MLSA analysis and phylogenomic analysis based on the concatenated alignment of 1,510 core orthologous proteins from strain B38^T^ and related *Psychrobacter* species supported its classification as a novel species.

The cellular fatty acid profile and the predominant respiratory quinone of strain B38ᵀ were consistent with those reported for closely related species within the genus *Psychrobacter*. Regarding to the phenotypic test performed, although strain B38^T^ showed the highest sequence identity with *P. alimentarius* JG 100^T^, several phenotypic differences were observed between these both strains as well as in comparison with other closest related species.

Based on the combined genomic, phylogenetic and chemotaxonomic results obtained, we concluded that strain B38^T^ represents a novel species, for which the name *Psychrobacter halotolerans* sp. nov. is proposed.

Strain B38^T^ belongs to a genus in which some strains have been proposed as PGP (Ma et al., 2009; Ma et al., 2010) and as biocontrol agents (Packiavathy et al., 2021; Reina et al., 2021). These bacteria tipically promote plant growth through multiple mechanisms, including the production of phytohormones and volatile organic compounds (Borker et al., 2024), siderophores (Sinha et al., 2019) and biosurfactants (Styczynski et al., 2022). In the present study, several *in vitro* PGP-related activities were detected for strain B38ᵀ, including acid phosphatase, DNase and phytase activities as well as siderophores production and the hydrolysis of Tween 20 and Tween 80. While these biochemical traits are qualitatively consistent with the observed growth promotion and stress mitigation *in planta*, they should be considered potential contributors rather than confirmed *in vivo* mechanisms. Thus, these findings provide a conceptual framework for future mechanistic studies.

To evaluate the effect of strain B38^T^ on lettuce tolerance to salinity stress, an *in vivo* experiment was conducted using lettuce plants grown under two salinity conditions (0 and 100 mM NaCl). Lettuce is considered a moderately salt-sensitive crop, with a salinity threshold ranging between 1.3 and 2 dS m^−1^ (Franzoni et al., 2022; Zuzunaga-Rosas et al., 2024). Our findings indicated that strain B38 ^T^ exhibited significant PGP properties, as evidenced by increased physiological parameters, such as root dry weight, root and shoot length and leaf area, under non-saline conditions. A similar PGP effect was also observed under salinity conditions, where all measured physiological parameters significantly increased with B38^T^ inoculation in comparison to non-inoculated plants. Overall, these results demonstrated that strain B38^T^ consistently enhances plant growth under both non-saline and saline conditions. Halotolerant bacteria from genera such as *Bacillus* (Oubaha et al., 2024), *Pseudomonas* (Kalleku et al., 2024) or *Halomonas* (Ould Ouali et al., 2024) have been widely reported as plant growth-promoting under salt stress, whereas the PGP potential of *Psychrobacter* in saline conditions remains largely unexplored.

The PGP effect of strain B38^T^ under saline stress was further supported by photosynthetic analyses. Specifically, B38^T^-inoculated plants exposed to 100 mM NaCl showed significantly higher Fv/Fm ratio than non-inoculated plants. The Fv/Fm ratio is widely considered as a robust indicator of the maximum quantum efficiency of PSII. The value of this parameter decreases in most plant species when they are exposed to stress conditions. Therefore, a reduction in Fv/Fm indicates damage to PSII and to photosynthetic apparatus (Adhikari et al., 2019; Makhtoum et al., 2023). Higher values of Fv/Fm following PGPB inoculation under saline conditions have been reported by ALKahtani et al. (2020), who observed higher Fv/Fm values in sweet pepper plants inoculated with *Bacillus thuringiensis* MH161336 under 34 and 68 mM NaCl compared to non-inoculated plants. This enhancement of plant growth and photosynthetic efficiency may contribute to the maintenance of lettuce yield and productivity under salt stress conditions.

After confirming the PGP activity of strain B38^T^ under both non-saline and saline conditions, we sought to elucidate the mechanisms underlying its protective effects against abiotic stress. Salt stress disrupts osmotic balance, ion homeostasis and induces oxidative damage in plants (Ikram et al., 2025). In response, plants increase the production of osmoprotectants to maintain cellular osmotic adjustment, alongside antioxidant molecules to detoxify ROS and alleviate oxidative stress (Singh et al., 2015).

Phenolic compounds constitute one of the principal plant defense mechanisms against oxidative stress. Their antioxidant activity is due to their redox properties, allowing them to scavenge free radicals effectively. The biosynthesis of phenolic compounds is commonly stimulated under biotic or abiotic stress conditions, including salinity (Hichem et al., 2009). In the present study, total free phenolic content significantly increased in B38^T^-inoculated plants as the concentration of NaCl increased. Notably, under 100 mM NaCl, plants inoculated with strain B38^T^ exhibited significant higher accumulation of phenolic compounds in comparison to non-inoculated plants. Previous studies such as Ayuso-Calles et al. (2020) observed that under 100 mM NaCl conditions lettuces inoculated with *Rhizobium laguerreae* HUTR05 showed higher levels of phenolic acids compared to non-inoculated plants. Additionally, Sánchez et al. (2023) reported increased phenolic levels in tomato plants under salt stress when co-inoculated with *Peribacillus castrilensis* N3 and EPS mauran. These findings indicate that strain B38^T^ enhances phenolic compounds biosynthesis under saline stress, thereby reducing oxidative damage in lettuce plants, as demonstrated by the significantly lower levels of malondialdehyde (MDA) in these conditions, being MDA a parameter widely used as an indicator of lipid peroxidation and oxidative stress in plants (Al-Turki et al., 2023). Similar effects have been reported in *Arabidopsis thaliana* and wheat. In *Arabidopsis*, Baek et al. (2020) observed no significant differences in MDA between plants inoculated with *Bacillus oryzicola* YC7007 and controls under non-saline conditions, but a 62.3% reduction in MDA at 100 mM NaCl in inoculated plants. In wheat, inoculation with *Bacillus mojavensis* I4 likewise significantly decreased MDA levels at 150 mM NaCl compared with non-inoculated plants (Ghazala et al., 2023). The reduction of MDA levels in lettuce under salt stress has been demonstrated both by İkiz et al. (2024), who reported a 41.5% decrease at 50 mM NaCl with a PGPR bioinoculant composed of *B. subtilis*, *B. megaterium*, and *Pseudomonas fluorescens*, and by Bai et al. (2023), who observed decreases of 24.56 and 39.91% at 100 and 150 mM NaCl, respectively, in lettuces inoculated with *B. velezensis* JB0319 compared to control plants.

Under salinity, plants synthesize compatible solutes to maintain low cellular water potential and ensure water uptake. Among these osmolytes, soluble sugars play a dual role as osmoprotectants and scavengers of ROS (Singh et al., 2022). Inoculation with PGPB have been shown to facilitate the accumulation of adequate levels of osmoprotectants in plants (Kang et al., 2015). In the present study, under saline stress, inoculation with strain B38^T^ significantly increased the concentration of total soluble sugars in lettuce compared to non-inoculated plants, a response similar to that observed in tomato, where application of the halotolerant bacterium *P. castrilensis* N3 and the exopolysaccharide (EPS) mauran enhanced soluble sugars, contributing to osmotic adjustment and protection of cell membranes under these conditions (Sánchez et al., 2023). Furthermore, Ghazala et al. (2023) indicated that under 150 mM NaCl wheat plants inoculated with *B. mojavensis* I4 have a higher concentration of soluble sugars in root and shoot than compared to non-inoculated plants. Similar results were obtained by Dai et al. (2025), that detected under 150 mM NaCl conditions an enhancement of soluble sugar content in rice plants inoculated with *Delftia tsuruhatensis* DYX29 in comparison to control plants. These findings suggest that strain B38^T^ may promote osmoprotectants accumulation and thereby contributes to improved salt stress tolerance.

Polyamine metabolism in plants has long been recognized as closely associated with development and stress responses. Putrescine, spermidine, and spermine are the most abundant polyamines in plants and play an important role in cell protection, particularly through the regulation of reactive oxygen species (ROS) homeostasis and osmotic adjustment (Singh et al., 2022). Changes in polyamine levels are commonly observed under salt stress, suggesting their involvement in plant adaptive responses to salinity (Mohammadi Alagoz et al., 2025). In this study, we quantified polyamine levels in lettuce leaves and found that salinity reduced putrescine and spermidine contents in both inoculated and non-inoculated plants, while spermine levels increased in both treatments. However, B38^T^ inoculation clearly modulated this response, as inoculated plants showed significantly lower levels of spermidine and spermine compared to non-inoculated controls, whereas putrescine remained unchanged. These results suggest that B38^T^ alters polyamine profiles during salt stress, potentially contributing to adaptive responses through the regulation of reactive oxygen species (ROS) homeostasis and osmotic balance. This interpretation is consistent with the higher photosynthetic rates and lower MDA levels observed in inoculated lettuce plants under these stress conditions. This response agrees with previous work showing that microbial inoculation can change polyamine metabolism under salt stress, particularly by altering the balance between spermine and spermidine (Kaushal, 2020). Previous studies in rice have reported that spermine accumulation in leaves does not alleviate salt-induced damage, indicating that its increase may be more a consequence of salt accumulation than a protective mechanism (Maiale et al., 2004). In this context, the lower spermine levels observed in B38^T^-inoculated plants under salinity may reflect reduced Na^+^ accumulation in leaves. Moreover, the exogenous application of spermidine in the irrigation solution has been shown to reduce Na^+^ transport from roots to shoots under saline conditions, helping to mitigate salt-induced damage (Zhu et al., 2006). In this context, we observed that the B38^T^ strain grown under saline conditions (1% NaCl) exhibited a 3-fold increase in spermidine production compared to control conditions (0% NaCl) (data not shown). In line with this finding, our laboratory is currently investigating the capacity of different PGP bacterial strains to synthesize and secrete spermidine into the plant root system. This potential mechanism may contribute to restricting Na^+^ translocation and modulating plant stress responses, thereby representing a promising strategy to enhance lettuce tolerance to salinity stress and further supporting the role of bacterial-derived polyamines in plant adaptation to adverse environmental conditions.

### 4.1 Description of *Psychrobacter halotolerans* sp. nov

*Psychrobacter halotolerans* (ha.lo.to′le.rans. Gr. n. *hals* salt; L. pres. part. *tolerans* tolerating; N.L. part. Adj. *halotolerans* referring to the ability of the organism to tolerate high salt concentrations).

Cells of this species are aerobic, Gram-negative, motile and coccoid to short rod-shape. On LB agar, colonies are small, creamy and circular. Growth occurs in the presence of 0–12% (w/v) NaCl (optimum 2.5% w/v), at pH varying from pH 6.0 to 10.0 (optimum pH 6.0–9.0), and at temperature ranging from 4 to 32° C (optimum 28° C). Catalase and oxidase activities are positive. According to Biolog GEN III microplates analyses, cells use the following substrates as sole carbon and energy sources: dextrin, D-fucose, L-fucose, glycyl-L-proline, L-alanine, L-arginine, L-aspartic acid, L-glutamic acid, L-histidine, L-serine, methyl pyruvate, D-lactic acid methyl ester, L-lactic acid, ɣ-keto-glutaric acid, D-malic acid, L-malic acid, Tween 40, ɣ-amino-butyric acid, β-hydroxyl-D, L-butyric acid, acetoacetic acid, propionic acid and acetic acid. All remaining substrates in the Biolog GEN III panel do not support, or only weakly support, growth. Based on Biolog GEN III sensitivity assays, growth occurs at pH 5.0 and pH 6.0, 1–8% (w/v) NaCl and 1% (w/v) sodium lactate. Cells are resistant to aztreonam, lincomycin, lithium chloride, nalidixic acid, potassium tellurite, rifamycin SV, sodium butyrate and tetrazolium blue, while growth is inhibited by all other compounds tested.

API 50 CH and API 20NE results obtained indicate that cells oxidize or assimilate esculin ferric citrate. Enzymatic activities detected using API 20NE and API ZYM include esterase (C8), lipase (C14), valine arylamidase and *α*-galactosidase, and weak activity observed for napthol-AS-BI-phosphohydrolase. Acids production from glucose is negative. Strain B38^T^ is positive for acid phosphatase, DNase and phytase, as well as for the hydrolysis of Tween 20 and Tween 80. Siderophore production and α hemolysin activity are also observed.

The main cellular fatty acids of strain B38^T^ are C_18:1_ ω9c and summed feature 3 (C_16:1_ ω7c/C_16:1_ ω6c). The major polar lipids identified is diphosphatidylglycerol, while the predominant respiratory quinone is ubiquinone-8 (Q-8).

The genome of *P. halotolerans* B38^T^ consists of a circular chromosome of 3.2 Mbp, G+C value of 43.81 mol% and 2,621 protein-coding genes. The whole genome sequence of *P. halotolerans* B38^T^ has been deposited in GeneBank under accession number JBDKWD000000000. The type strain is B38^T^ (= CECT31210 = LMG33901), isolated from the phyllopshere of the halophyte plant *Salicornia hispanica*.

## 5 Conclusion

In this study, we characterized a novel *Psychrobacter* species isolated from the phyllopshere of the halophyte plant *Salicornia hispanica*. Using a polyphasic taxonomic approach, we demonstrate that strain B38^T^ represents a novel species. Additionally, this study highlights the potential of *P. halotolerans* B38^T^ as plant growth-promoting bacteria (PGPB), given its growth-promoting activity in lettuce plants under both non-saline and saline conditions, as well as its ability to confer salt stress tolerance in lettuce by enhancing osmoprotectants production and reducing lipid peroxidation and oxidative damage. These characteristics suggest that *P. halotolerans* has great biotechnological potential as an inducer of salinity tolerance in the agricultural sector.

## Data Availability

The accession numbers are available and the data have been deposited. The corresponding identifiers are as follows: JBDKWD000000000 https://www.ncbi.nlm.nih.gov/nuccore/JBDKWD000000000GCA_039680845, https://www.ncbi.nlm.nih.gov/datasets/genome/GCA_039680845.1/SAMN41148270, https://www.ncbi.nlm.nih.gov/biosample/?term=SAMN41148270PRJNA1106776, https://www.ncbi.nlm.nih.gov/bioproject/1106776GCA_039680845, https://www.ncbi.nlm.nih.gov/datasets/genome/GCF_039680845.1/.

## Footnotes

1
https://sourceforge.net/project/bbmap/

2
http://enve-omics.ce.gatech.edu/aai/

2
http://ggdc.dsmz.de/

3
https://www.ibm.com/docs/en/spss-statistics/28.0.0

## Supplementary material

The Supplementary material for this article can be found online: https://www.frontiersin.org/articles/10.3389/fmicb.2026.1856557/full#supplementary-material
