# Supplementary figures and images for: Psychrobacter halotolerans sp. nov., a halotolerant plant growth-promoting bacterium that enhances lettuce tolerance to salt stress
Source: Front Microbiol. 2026 Jun 29;17:1856557. doi: 10.3389/fmicb.2026.1856557 (PMC13357991; doi:10.3389/fmicb.2026.1856557)

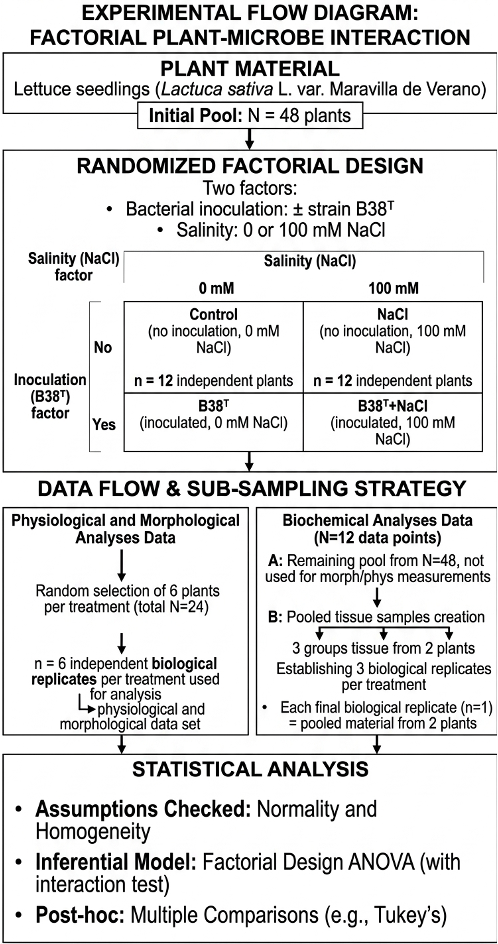

Supplement: Supplementary file 1 [file Image_1.png]

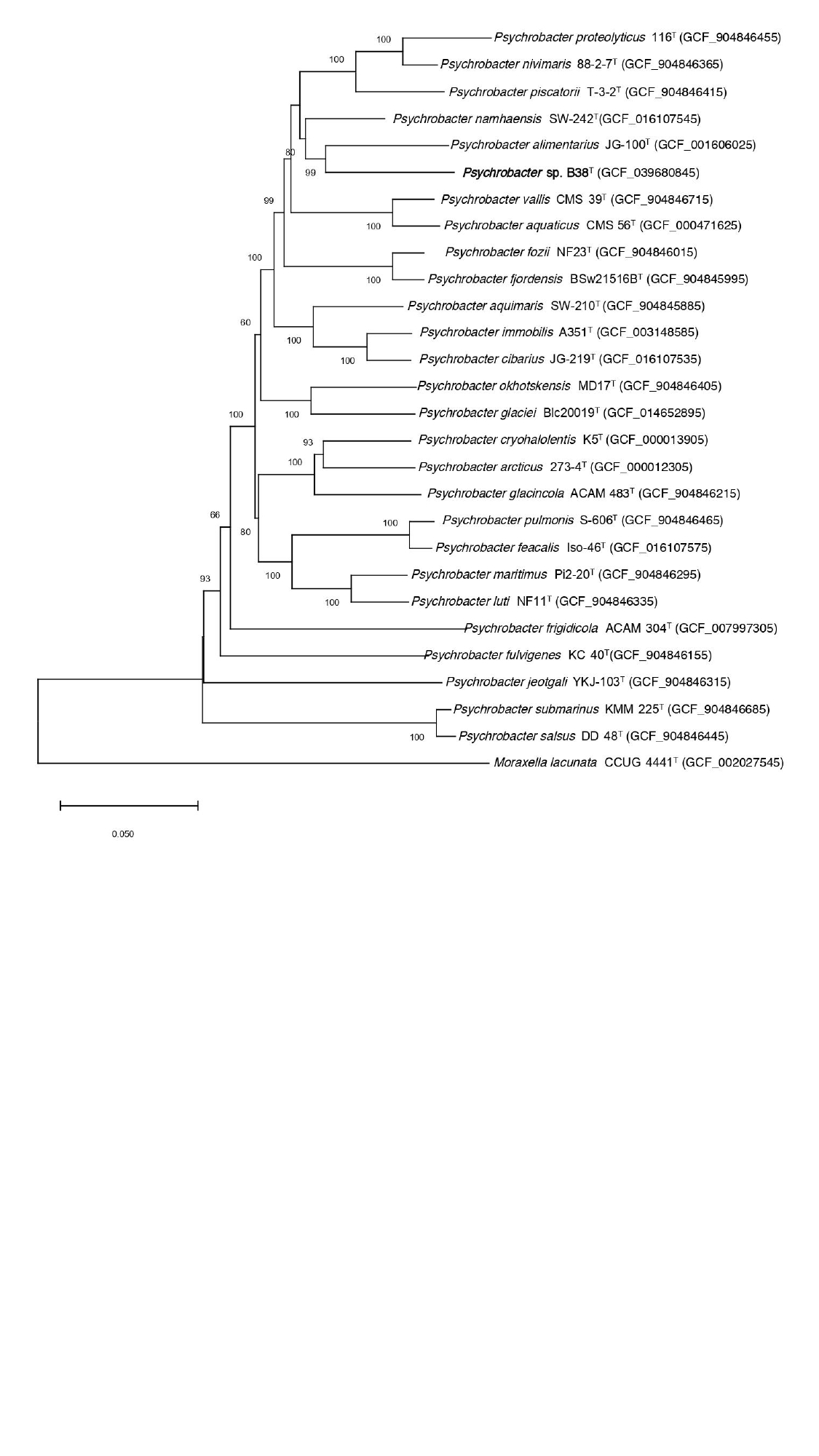

Supplement: Supplementary file 2 [file Image_2.jpeg]

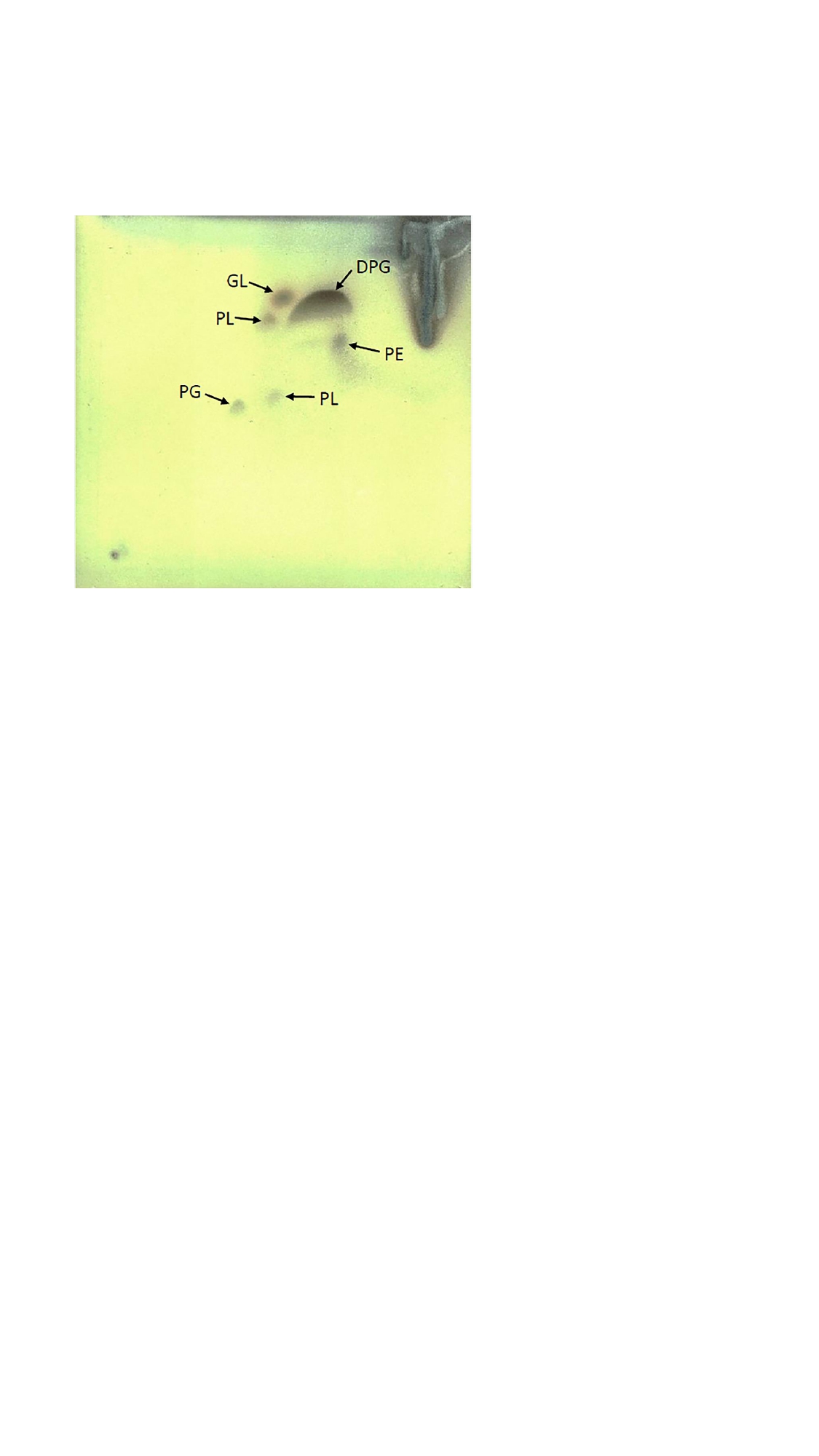

Supplement: Supplementary file 3 [file Image_3.jpeg]

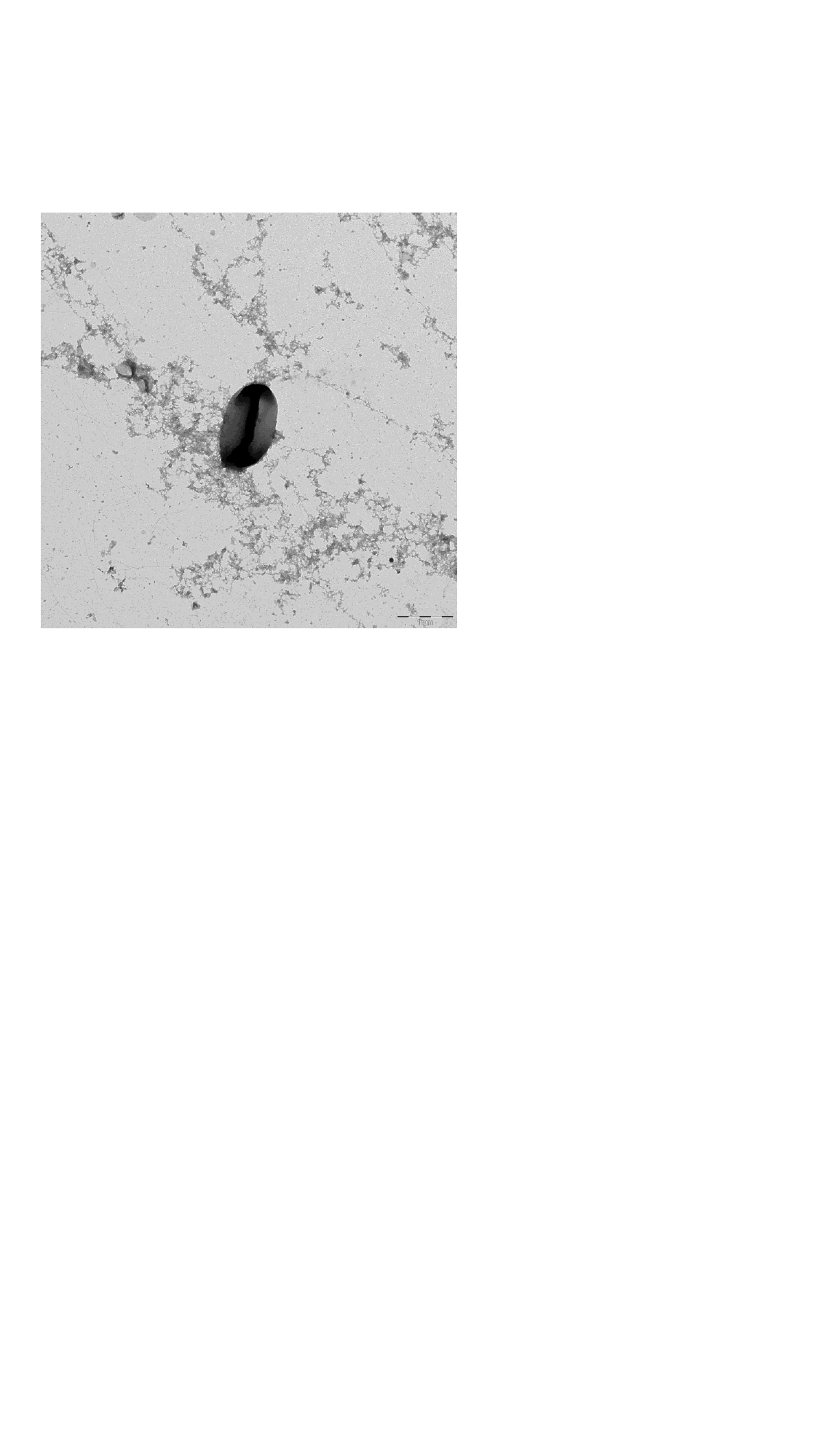

Supplement: Supplementary file 4 [file Image_4.jpeg]
