# Supplementary material for: Psychrobacter halotolerans sp. nov., a halotolerant plant growth-promoting bacterium that enhances lettuce tolerance to salt stress
Source: Front Microbiol. 2026 Jun 29;17:1856557. doi: 10.3389/fmicb.2026.1856557 (PMC13357991; doi:10.3389/fmicb.2026.1856557)
Supplement: Supplementary file 8 [file Table_4.docx]

Supplementary Material

*Psychrobacter halotolerans* sp. nov., a halotolerant plant growth-promoting bacterium that enhance lettuce tolerance to salt stress

Patricia Sánchez^1^, Fernando Martínez-Checa^1,3^, Francisco Palma^2^, Inmaculada Llamas^1,3*^, Inmaculada Sampedro^1,3^

^1^ Department of Microbiology, Faculty of Pharmacy, University of Granada, Granada, Spain

^2^ Department of Plant Physiology, Science Faculty, University of Granada, Granada, Spain

^3^ Biomedical Research Centre (CIBM), Biotechnology Institute, Granda, Spain

*** Correspondence:**Inmaculada Llamas
illamas@ugr.es

# Supplementary Figures and Tables

## Supplementary Figures

**Supplementary Figure S1.** A CONSORT-style flow diagram illustrating the allocation of plants to each treatment and their use in the different analyses.

**Supplementary Figure S2**. Phylogenetic tree reconstructed according to the neighbor-joining method based on the concatenation of the genes 16S rRNA, *rpo*B, *rpo*D and *gyr*B of the strain B38^T^ (bold) and *Psychrobacter* related species. The GenBank/EMBL/DDBJ accession number of each sequence is shown in parenthesis. Bootstrap values are expressed as percentages of 1,000 replications, and those over 50% are shown at branch points. Bar−0.010 substitutions per nucleotide position. The strain *M. lacunata* CCUG 4441^T^ was used as an outgroup.

**Supplementary Figure S3**. Polar lipid profile of strain B38^T^ determined after two-dimensional TLC using molybdatophophoric acid. DPG: diphosphatidylglycerol, PE: phosphatidylethanolamine; PG: phosphatidylglycerol, GL: glycolipid, PL: phospholipid.

**Supplementary Figure S4**. Image obtained by transmission electron microscopy (TEM) of the strain B38^T^.

## Supplementary Tables

**Supplementary Table S1**. Genome sequence similarity between strain B38^T^ and genome sequences of closely related type strains with available genomes.

**Supplementary Table S2**. Phenotypic characterization of the strain B38^T^ described in this study and their closest species.

**Supplementary Table S3**. Phenotypic characteristic of the strain B38^T^ .
